# Chronic Periodontitis and the Potential Likelihood of Gastric Cancer: A Nested Case-Control Study in the Korean Population Utilizing a National Health Sample Cohort

**DOI:** 10.3390/cancers15153974

**Published:** 2023-08-04

**Authors:** Mi Jung Kwon, Ho Suk Kang, Min-Jeong Kim, Nan Young Kim, Hyo Geun Choi, Hyun Lim

**Affiliations:** 1 Department of Pathology, Hallym University Sacred Heart Hospital, Hallym University College of Medicine, Anyang 14068, Republic of Korea; mulank@hanmail.net; 2 Division of Gastroenterology, Department of Internal Medicine, Hallym University Sacred Heart Hospital, Hallym University College of Medicine, Anyang 14068, Republic of Korea; hskang76@hallym.or.kr; 3 Department of Radiology, Hallym University Sacred Heart Hospital, Hallym University College of Medicine, Anyang 14068, Republic of Korea; drkmj@hallym.or.kr; 4 Hallym Institute of Translational Genomics and Bioinformatics, Hallym University Medical Center, Anyang 14068, Republic of Korea; honeyny78@gmail.com; 5 Suseo Seoul E.N.T. Clinic and MD Analytics, Seoul 06349, Republic of Korea; mdanalytics@naver.com

**Keywords:** gastric cancer, chronic periodontitis, nested case–control study, national healthcare data

## Abstract

**Simple Summary:**

This research investigates the potential link between gum disease and stomach cancer in the Korean population. In this study, we analyzed data from over 10,000 people with gastric cancer and 40,000 healthy individuals. The results indicate that individuals who had gum disease in the past one to two years were more likely to develop stomach cancer. This association was consistently observed in different groups, such as men under 65 years old. The study emphasizes the importance of regular screening for stomach cancer, particularly for those at high risk, such as individuals with a history of gum disease. These findings contribute to our understanding of the connection between oral health and stomach cancer and may guide future preventive measures.

**Abstract:**

There is limited information regarding the potential association between chronic periodontitis (CP) and gastric cancer, especially in the Korean population. This study aimed to explore this relationship. This nested case–control study analyzed data from 10,174 patients with gastric cancer and 40,696 controls from the Korean National Health Insurance Service–National Sample Cohort using propensity score matching. Standardized differences were used to compare baseline characteristics between study groups. Logistic regression analyses adjusted for confounders were conducted to assess the association between history of CP and gastric cancer occurrence. CP histories and comprehensive subgroup analyses in the 1- and 2-year periods preceding the index date were evaluated. Individuals with a history of CP within the 1-year and 2-year periods showed an increased likelihood of developing gastric cancer. Subgroup analyses consistently supported these findings in male participants aged <65 years and individuals with various income levels or living in residential areas. However, no significant associations were observed among participants aged ≥65 years. In conclusion, CP may be a potential risk factor for gastric cancer development in the Korean population. Regular screening for gastric cancer may be necessary for high-risk individuals, specifically men aged <65 years with a history of CP.

## 1. Introduction

Gastric cancer is the fifth most prevalent cancer and a significant cause of cancer-related mortality in Korea [1], which is among the regions with the highest risk of stomach cancer worldwide [1]. Gastric cancer develops through a complex process involving multiple factors and stages [2], the key contributors of which include Helicobacter pylori infection, geographic variations, and lifestyle choices [2]. Over the past two decades, the implementation of a mandatory national endoscopic screening program for gastric cancer, conducted every two years for adults above 40 years of age, has contributed to a reduction in gastric cancer mortality [3]. Despite ongoing efforts, gastric cancer remains the most commonly diagnosed malignancy among adult men in Korea, and its incidence continues to increase with age in both males and females in Korea [1]. These trends highlight that relying solely on screening measures may not be sufficient to fully control the incidence of stomach cancer; hence, identifying modifiable risk factors associated with gastric cancer is crucial for establishing a foundation for fundamental prevention approaches.

Chronic periodontitis (CP) is a prevalent inflammatory condition that primarily affects the gums and is commonly observed in adults aged 30 years and older, with higher rates observed in individuals aged over 50 years [4]. In this condition, there is an infiltration of immune-inflammatory cells into the deep structures of the periodontium, causing progressive devastation of the periodontal tissues and underlying alveolar bone, which is responsible for supporting the teeth [5,6]. This process contributes significantly to tooth loss in adults [4], significantly affecting their overall quality of life [5,6]. The potential connection between CP and various systemic disorders, including cancer, has attracted considerable attention [7]. Epidemiological studies worldwide have consistently reported a potential link between CP and an elevated risk of gastric cancer [8,9,10,11,12,13,14]. The foundation of this connection lies in the substantial roles of inflammation and dysbiosis observed under both conditions [15]. Local chronic inflammation may be a key factor underlying this association, as multiple studies have established a correlation between chronic inflammation and various gastrointestinal cancers such as gastric cancer [16]. This correlation raises concerns regarding the potential risk of developing comorbid gastric cancer, particularly among elderly individuals, given the high prevalence of gastric cancer in Korea [1]. However, there is insufficient information regarding this association, particularly among the Korean population.

In contrast with these positive findings, previous studies have reported the null relevance of CP in gastric cancer [17,18,19]. These studies often had imbalanced sample sizes between the study and comparison groups and included cohorts with different demographics, such as age, sex, ethnicity, education level, or income level [18,19]. Furthermore, some studies included a small number of gastric cancer cases [18,19]. While a meta-analysis that included various gastrointestinal cancers observed a significant association between CP, including tooth loss, and overall gastrointestinal cancer [20], the specific impact of CP on the incidence of gastric cancer has not been thoroughly explored, as the primary focus of the meta-analysis was not solely on gastric cancer [20]. Another meta-analysis specifically investigating gastric cancer found a notable link between CP and gastric cancer, which was limited by a considerable degree of heterogeneity among the included studies [21]. To accurately assess the potential impact of CP on the risk of developing gastric cancer, further research using validated and well-balanced large-scale cohort data that specifically focuses on gastric cancer is required.

We hypothesized that a history of CP could negatively influence the development of gastric cancer and that there might be specific risk factors associated with CP that could predict the occurrence of gastric cancer in the Korean population. This study aimed to explore these relationships by conducting a nationwide cohort study using a meticulously matched nested case–control design and comprehensive subgroup analyses to examine the potential effects of CP on the development of stomach cancer.

## 2. Materials and Methods

The ethics committee of Hallym University approved this study under the reference number 2022-10-008. Written informed consent was not required, as the analysis was conducted using anonymous data. The data sources used in this study were provided by the Korean National Health Insurance Service-National Sample Cohort (KNHIS-NSC) [22]. This database comprised 1,108,369 participants and 219,673,817 medical claims codes recorded between January 2002 and December 2019. The KNHIS-NSC was generated by the Korea National Health Insurance Service, which applied a systematic sampling procedure to create a prototypical sample of 1,025,340 people in 2002, accounting for approximately 2.2% of all Korean citizens. These individuals were followed up for 17 years, until 2019. Further details regarding the representativeness of the data and the cohort can be found elsewhere [22].

To investigate the relationship between the history of subjects and the presence or absence of exposure in outcome status, a nested case–control study design was deemed appropriate [23]. The National Health Insurance database employs the International Classification of Diseases, 10th revision (ICD-10) codes as a standardized reference for disease diagnosis and healthcare information organization within the database. In this study, participants who were newly diagnosed with stomach cancer were initially selected based on their diagnosis of gastric cancer (ICD-10 codes: C16.0–C16.9) and the presence of a special claim code indicating serious cancer (V193 or V194) between 2005 and 2019 (n = 10,174). A special claims code indicating a serious illness that makes cancer patients eligible for payment reduction has been implemented in the national healthcare service since 2005. Individuals not diagnosed with gastric cancer from 2005 to 2019 were included in the control group (n = 1,098,195). Individuals in the control group who were diagnosed with stomach cancer at least once (n = 2412) were excluded from the study.

**Figure 1:**
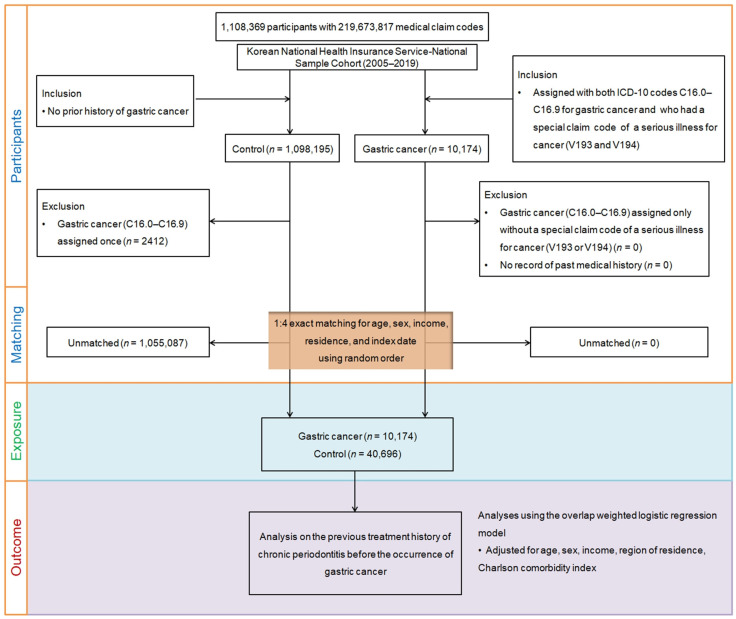
A schematic illustration of the participant selection process employed in this study. Out of the initial cohort of 1,108,369 participants, 10,174 individuals with gastric cancer were matched with 40,696 control participants based on age, sex, income, and region of residence.

To minimize disparities in baseline demographic and clinical characteristics between the gastric cancer and counterpart groups, propensity score matching was performed [24,25]. The matching process entailed pairing participants with gastric cancer with control participants who exhibited similar propensity scores, which were based on 4 covariates (sex, age, income, and region of residence) [24,25]. The index date for each patient diagnosed with stomach cancer was established as the day when both the ICD-10 codes for stomach cancer and the special claim code indicating a serious cancer illness (V193 or V194) were electronically recorded in the health care datasets [25]. For the control group, the index date was defined as the index date of the matched patients with stomach cancer [25]. As a result, each matched patient and control group shared an identical index date. During matching, 1,055,087 participants were excluded. Ultimately, 10,174 stomach cancer patients were matched in a 1:4 ratio with 40,696 controls (Figure 1). We then surveyed patients with a history of CP in both 1-year and 2-year periods before the index date in both groups.

### 2.1. Exposure (Chronic Periodontitis)

To ensure the accuracy of the analysis and eliminate false-positive cases, only participants who were treated for CP by dentists and diagnosed according to the ICD-10 code (K05.3) were included in the study [26]. The number of CP treatments received by each participant was documented until the day preceding both the 1-year and 2-year periods prior to the index date, and a meticulous approach was adopted to enhance the reliability of the association between CP and the outcomes under investigation.

### 2.2. Outcome (Gastric Cancer)

To maintain the precision of the analysis and avoid false-positive cases, stomach cancer cases were determined employing specific ICD-10 codes (C16.0–C16.9) allocated as the diagnosis [malignant neoplasm of the cardia (C16.0), fundus (C16.1), body (C16.2), pyloric antrum (C16.3), pylorus (C16.4), lesser curvature, unspecified (C16.5), greater curvature, unspecified (C16.6), overlapping sites of the stomach (C16.8), and the stomach, unspecified (C16.9)] [25]. Additionally, a special claims code indicating serious cancer illness (V193 or V194) was utilized to confirm eligibility for reduced payments from the national healthcare service.

### 2.3. Covariates

Participants were classified into 10 age categories, each illustrating a 5-year gap, and were distributed into five income categories, ranging from class 1 (lowest income) to 5 (highest income). Residential locations were categorized into 16 groups based on administrative districts and subsequently reorganized into urban or rural areas [26]. The Charlson Comorbidity Index (CCI) is a commonly employed tool for assessing the overall burden of comorbid conditions, calculated as a sum score ranging from 0 to 29, considering 17 potential comorbidities [27,28]. CCI score was included as a covariate in the analysis, excluding cases of gastric cancer. This was performed to consider the potential influence of comorbidities on the development of gastric cancer [28].

### 2.4. Statistical Analyses

Standardized differences were applied to compare baseline features between the study groups. An absolute standardized difference of less than 0.20 was considered indicative of achieving satisfactory balance [29]. The propensity score was computed using multivariate logistic regression, incorporating all relevant covariates. In the overlap weighting approach, participants with gastric cancer were weighted based on the propensity score probability, while control participants were weighted based on the probability of 1 minus the propensity score. This overlap weighting, ranging between 0 and 1, was utilized to achieve optimal balance and enhance the precision of the analyses [30,31,32]. The analysis of the overlap-weighted odds ratios (ORs) involved computing odds ratios with 95% confidence intervals (CIs) for gastric cancer in relation to the number of treatments for chronic periodontitis (per 10 treatments), both in the crude (unadjusted) and overlap weighted models (adjusted for age, sex, income, residence area, and CCI). Additionally, subgroup analyses were conducted based on age, sex, income, and region of residence.

Statistical analyses were conducted using SAS version 9.4 (SAS Institute Inc., Cary, NC, USA). Two-tailed analyses were performed, and statistical significance was determined at a significance level of *p* < 0.05.

## 3. Results

Table 1 provides the demographic characteristics of 10,174 patients diagnosed with gastric cancer, along with a comparison group of 40,696 individuals at baseline. The table includes data both before and after conducting overlap weighting adjustments for propensity score matching. Prior to adjustment, the covariates age, residence, sex, and income exhibited a standardized difference of 0.00, indicating no significant disparities between the gastric cancer and control groups. Other basic characteristics, such as the number of CP treatments for 1 year and 2 years before the index date, were not significantly different (both standardized differences = 0.01). However, the gastric cancer group had higher CCI scores than the control group (standardized difference = 0.67). After implementing overlap-weighting adjustments, these discrepancies were notably reduced, with each covariate achieving a standardized difference of <0.2. This indicates a well-balanced distribution of demographic attributes between the gastric cancer and control groups.

To ensure the reliability of our findings, we conducted a rigorous analysis by evaluating CP histories at both 1-year and 2-year periods before the index date (Table 2). The results consistently showed a meaningful positive relationship between a history of CP and incident gastric cancer. The OR 1 year was 1.31 (95% CI = 1.15–1.49, *p* < 0.001), indicating a 31% enhanced probability of gastric cancer. Similarly, for 2 years, the OR was 1.24 (95% CI = 1.14–1.35, *p* < 0.001), suggesting a 24% elevated chance.

Subgroup analyses were conducted to examine associations within specific demographic categories. In the evaluation of CP history within the 1-year period prior to the index date, a significant association persisted among participants aged <65 years, males, and individuals across different income levels or residence areas. However, no significant association was observed among participants aged ≥65 years.

Similarly, in the evaluation of CP history within the 2-year period before the index date, consistent findings were observed across subgroups. CP was significantly associated with gastric cancer in participants aged <65 years, males, females, those with low or high income, and both urban and rural residents. However, similar to the 1-year period analysis, no significant association was found in the age ≥65 years subgroup.

## 4. Discussion

In this extensive nationwide cohort study, we conducted a rigorous evaluation by examining two distinct periods to establish a reliable association between CP and gastric cancer. These results consistently indicated that individuals who had previously experienced CP had a slightly but significantly elevated probability of developing gastric cancer. Specifically, within 1 year preceding the index date, there was a 30% increased likelihood of gastric cancer development (95% CI = 1.15–1.49), while the increased likelihood was 24% (95% CI = 1.14–1.35) within 2 years prior to the index date, which provides further support for the strong relationship between the two conditions. This association was more pronounced in males aged <65 years, regardless of income or residence. However, no such association was observed among individuals aged ≥65 years. Based on our study findings, regular screening for gastric cancer may be particularly necessary for high-risk individuals, specifically men aged <65 years with a history of CP.

Our findings appear to align with the results of previous studies conducted in various countries, including China [8], Finland [9], Iran [10], Japan [11], Sweden [13], the USA [14], and Turkey [12], which may further strengthen the evidence supporting a strong relationship between CP and gastric cancer. In a substantial prospective cohort study conducted in a rural area of China, involving 28,868 individuals aged 40–69, it was discovered that tooth loss due to CP was significantly associated with an 80% increased risk (95% CI = 1.1–3.0) of gastric cancer, particularly in non-cardiac locations [8]. In another prospective cohort study conducted in Finland that included male smokers (n = 29,124), edentulous individuals with CP were found to have a significant (1.65-fold) increased risk of gastric noncardia adenocarcinoma (95% CI = 1.09–2.49) [9]; however, the participants self-reported their dental history in questionnaires, which may introduce recall bias, and the exclusive enrollment of males may limit the study’s generalizability to the overall population [9]. In another study conducted in the USA, which combined two independent studies involving both male and female health professionals, a history of periodontal disease was found to be associated with a 52% greater risk of stomach cancer [14]; however, health-professional-only cohorts may cause selection bias [14]. Similarly, a case–control study conducted in Hokkaido, Japan, including 242 gastric cancer cases and 484 matched controls, revealed that the CP accompanied by loss of more than 10 teeth showed a 1.73-times aggravated likelihood of developing stomach cancer (95% CI = 1.23–2.43) [11]. The generalizability of previous studies on the association between CP and gastric cancer may be diminished due to their reliance on community-based cohorts [8,10,11,12,13], which may not fully represent the entire population. Our study used a nationwide organized dataset covering a wide range of participants, which enabled us to minimize confounding effects and enhance the generalizability of our findings. The increased probability of gastric cancer associated with CP was consistently reproduced in our study, which involved a robust sample size with a balanced distribution of 10,174 patients with gastric cancer matched with 40,696 control participants.

Throughout our analysis, we consistently found a positive association indicating an increased likelihood of developing gastric cancer in CP individuals aged <65 years and in men, irrespective of income or residence, highlighting the importance of age and sex considerations in the association between CP and gastric cancer. These results remained consistent across the two different time periods studied. The association between CP and gastric cancer appears to vary based on different age groups. We observed a 38% higher likelihood of the occurrence of stomach cancer in patients under the age of 65 (95% CI = 1.22–1.55), while no such relevance was found in the older age group of over 65 years (OR 1.09; 95% CI = 0.96–1.23). The fact that there is an exclusively positive association between age <65 years in CP patients and incident gastric cancer is intriguing, especially considering the higher prevalence of periodontitis in subjects aged 70 to 81 years compared to those aged 50 to 59 years [33]. The incidence of CP is more prominent in older adults aged 65 years or above, with approximately two-thirds (68%) of individuals in this age group being affected [33]. Despite this, our findings seem to align with those of two previous studies that also demonstrated an elevated gastric cancer risk in younger individuals <65 years with CP [8,13]. A Swedish study indicated a 4.24-fold increased risk for individuals aged 50 (95% CI = 1.83–9.80), which gradually decreased by approximately 4% per year, resulting in a diminished magnitude of 1.54 (95% CI = 0.90–2.64) for individuals aged 75 [13]. Similarly, a Chinese study observed a decreased relative risk of gastric cancer with increasing age among participants with CP, with the strongest association observed in individuals aged <50 years [8]. The authors noted that in this region, a considerable number of individuals experience tooth loss at an early stage of life [8]. Coincidentally, this population also exhibits exceptionally high rates of esophageal and gastric cardia cancer [8], suggesting that the presence of CP in younger individuals could potentially serve as an early indicator of upper gastrointestinal cancer. Other studies from Finland and Iran also identified age as a significant factor influencing the association between the two diseases [9,10].

Our data consistently revealed a positive association in men, regardless of the two different time periods considered. This finding suggests that men and women with CP may indeed have differing degrees of risk of developing gastric cancer. Specifically, in the period from the index date to before the 2-year period, females showed a 20% higher likelihood of stomach cancer occurrence (95% CI = 1.02–1.42), whereas no statistically significant relevance was found in the period from the index date to before the 1-year period (OR 1.25; 95% CI = 0.97–1.62). Considering the male gender as an acknowledged risk factor in both CP and gastric cancer [1,4], our results may suggest that a subset of women who experience CP for a long-term period of over 2 years might be associated with a higher likelihood of developing gastric cancer.

Disparities in the prevalence and severity of periodontal disease have been linked to socioeconomic factors, including education and income [34]. Epidemiological reports consistently demonstrate an inverse relationship between periodontal disease and education and income, even after controlling for age and gender [34,35]. Additionally, socioeconomic status is known to be a risk factor for gastric cancer [36], with individuals of lower socioeconomic status facing a higher risk of gastric cancer and poorer survival rates [36,37]. As a result, it has been suggested that socioeconomic status or residential areas could potentially confound the interpretation of the association between CP and gastric cancer. In our present study, we made a notable observation of an increased likelihood of developing gastric cancer in individuals with CP who were men and aged <65 years, regardless of income or residential area. This association remained significant even after adjusting for potential confounding factors. These findings indicate that factors related to socioeconomic status or residential areas do not fully explain the association between CP and gastric cancer in our study population. There is currently a lack of studies that have conducted comprehensive subgroup analyses considering sex, income, or residential area to determine the probability of developing gastric cancer shown in our study. It may be advisable to conduct clinical risk evaluations for gastric cancer in individuals with CP, particularly in men aged <65 years, within the Korean population. Further research and comprehensive subgroup analyses are warranted to fully elucidate the potential factors influencing this association and to develop targeted preventive strategies for at-risk populations.

In contrast, several studies have reported conflicting outcomes regarding the relationship between CP and the incidence of gastric cancer. These studies, conducted in Japan [38], Taiwan [17], and the USA [18,19], did not specifically focus on gastric cancer but included it as part of a broader range of cancers [17,18,19,38]. Therefore, the number of stomach cancer cases in these studies may have been insufficient for a comprehensive evaluation. The inconsistencies in research outcomes could be attributed to various factors, including varying definitions of CP, data collection methods (e.g., self-reported cancer diagnosis or CP history), variations in study cohort characteristics, differences in study designs and statistical analysis methods, geographic variations between regions for the incidence rates of gastric cancer, chance discoveries, discrepancies in exposure assessment, and variations in the adjustment for confounding factors [10,14,18,19]. A meta-analytic study focusing solely on gastric cancer identified a stronger association between CP and stomach cancer among the Asian population (OR 1.35; 95% CI = 1.14–1.59) than the European (OR 1.30; 95% CI = 0.75–2.26) or American populations (OR 0.96; 95% CI = 0.56–1.64) [21]. This suggests that the effects of CP on gastric cancer may vary depending on the geographic region, potentially owing to differences in gastric cancer incidence rates.

However, the mechanisms linking CP to subsequent gastric cancer remain unknown. Several hypotheses, including chronic inflammation, oral bacterial translocation, immune response, and alterations in the oral and gut microbiomes, have been suggested to explain this connection [7,39,40]. First, the release of proinflammatory cytokines and inflammatory mediators from the inflamed gums of CP can lead to systemic inflammation throughout the body [7,39]. The prolonged presence of inflammatory molecules may promote cancer development and progression of gastric cancer [16]. Elevated levels of inflammatory markers, including interleukin-1 beta (IL-1β), C-reactive protein (CRP), and tumor necrosis factor-alpha (TNF-β), have been identified in CP and gastric cancer [41,42,43,44]. Second, bacteria present in the oral cavity, which are associated with CP, can potentially migrate or translocate from the mouth to the stomach [16,40]. The translocation of pathogens from the oral cavity to the stomach can stimulate an immune response, leading to sustained inflammation and consequent damage to stomach tissues [15] or the potential tumorigenesis of gastric cancer [16,40]. Certain oral bacteria, including *Streptococcus anginosus*, *Tannerella forsythia*, and *Peptostreptococcus stomatis*, have been detected in stomach cancers and are associated with an increased risk of developing gastric cancer [16]. Finally, CP can disturb the equilibrium of the oral microbiome, leading to an imbalance in the bacterial composition within the oral cavity [45]. Research indicates that modifications to the oral microbiome can affect the composition of the gut microbiome [45,46]. These alterations in the gut microbiome are associated with the development of gastrointestinal cancers [47]. Thus, it is plausible that an imbalance in the gut microbiota resulting from CP could play a role in the pathogenesis of gastric cancer [40].

The strength of this study is that the results could be drawn from a very large cohort that represents the entire Korean population. The NHIS-NSC database provides extensive access to participants’ medical histories from healthcare facilities nationwide, thus improving the generalizability and accuracy of the study outcomes. Through overlap-weighted propensity score matching, we achieved a precise pairing of patients (n = 10,174) and control (n = 40,696). Although CP is more prevalent in men, the elderly, individuals with low socioeconomic status, rural residents, and those with a high CCI score [12,17,19], our study successfully achieved a balanced distribution of age, sex, income, residence, and CCI score among the participants, which effectively minimized selection bias and created study groups that closely resembled those observed in randomized clinical trials [23]. Demographic heterogeneity among participants may have influenced the magnitude of the associations observed between the original characteristics of the research groups [48]. 

Our study has some limitations. First, the retrospective observational design prevented us from establishing a definitive causal relationship between CP and stomach cancer. Second, our study specifically focused on the Korean population and relied on diagnosis codes from the Korean Health Insurance data, which may have led to the exclusion of unmeasured confounding variables not being accounted for and may have hindered the extrapolation of our results to other demographic groups. Finally, the NHIS-NSC database does not contain detailed information on the severity of CP, *Helicobacter pylori* infection status, stage of gastric cancer, histology, differentiation, family history, genetic data, or lifestyle factors. Therefore, the analysis conducted in this study did not account for the missing data.

## 5. Conclusions

This study provides the initial evidence of a potential association between CP and stomach cancer in the Korean population. Specifically, men aged <65 years with a history of CP may be at higher risk, and regular clinical screening for gastric cancer may be necessary. However, further research is necessary to validate and expand upon these findings and to investigate the mechanisms underlying this association.

## Figures and Tables

**Table 1:** General characteristics of participants after and before propensity score overlap weighting adjustment.

Characteristics		Before PS Overlap Weighting Adjustment			After PS Overlap Weighting Adjustment		
		Gastric Cancer	Control	Standardized Difference	Gastric Cancer	Control	Standardized Difference
Age (y), n (%)				0.00			0.00
	5–9	1 (0.01)	4 (0.01)		1 (0.01)	1 (0.01)	
	10–14	3 (0.03)	12 (0.03)		2 (0.02)	2 (0.02)	
	20–24	1 (0.01)	4 (0.01)		1 (0.01)	1 (0.01)	
	25–29	19 (0.19)	76 (0.19)		13 (0.17)	13 (0.17)	
	30–34	95 (0.93)	380 (0.93)		63 (0.85)	63 (0.85)	
	35–39	205 (2.01)	820 (2.01)		140 (1.91)	140 (1.91)	
	40–44	466 (4.58)	1864 (4.58)		334 (4.56)	334 (4.56)	
	45–49	711 (6.99)	2844 (6.99)		505 (6.89)	505 (6.89)	
	50–54	994 (9.77)	3976 (9.77)		701 (9.56)	701 (9.56)	
	55–59	1197 (11.77)	4788 (11.77)		856 (11.68)	856 (11.68)	
	60–64	1449 (14.24)	5796 (14.24)		1041 (14.21)	1041 (14.21)	
	65–69	1463 (14.38)	5852 (14.38)		1058 (14.43)	1058 (14.43)	
	70–74	1490 (14.65)	5960 (14.65)		1085 (14.80)	1085 (14.80)	
	75–79	1071 (10.53)	4284 (10.53)		784 (10.69)	784 (10.69)	
	80–84	693 (6.81)	2772 (6.81)		512 (6.99)	512 (6.99)	
	85+	316 (3.11)	1264 (3.11)		235 (3.20)	235 (3.20)	
Sex, n (%)				0.00			0.00
	Male	6834 (67.17)	27,336 (67.17)		4927 (67.23)	4927 (67.23)	
	Female	3340 (32.83)	13,360 (32.83)		2401 (32.77)	2401 (32.77)	
Income, n (%)				0.00			0.00
	1 (lowest)	1959 (19.25)	7836 (19.25)		1399 (19.09)	1399 (19.09)	
	2	1260 (12.38)	5040 (12.38)		896 (12.23)	896 (12.23)	
	3	1621 (15.93)	6484 (15.93)		1165 (15.89)	1165 (15.89)	
	4	2144 (21.07)	8576 (21.07)		1535 (20.95)	1535 (20.95)	
	5 (highest)	3190 (31.35)	12,760 (31.35)		2334 (31.84)	2334 (31.84)	
Region of residence, n (%)				0.00			0.00
	Urban	4310 (42.36)	17,240 (42.36)		3107 (42.40)	3107 (42.40)	
	Rural	5864 (57.64)	23,456 (57.64)		4221 (57.60)	4221 (57.60)	
CCI score, mean (SD)		2.40 (2.70)	0.92 (1.58)	0.67	1.71 (1.88)	1.71 (0.97)	0.00
The number of CP treatments for 1 year before index date, mean (SD)		0.52 (1.31)	0.51 (1.36)	0.01	0.54 (1.13)	0.49 (0.56)	0.05
The number of CP treatments for 2 year before index date, mean (SD)		1.00 (2.09)	0.98 (2.08)	0.01	1.03 (1.78)	0.94 (0.86)	0.06

Abbreviations: PS—propensity score; CCI—Charlson Comorbidity Index; SD—standard deviation; CP—chronic periodontitis.

**Table 2:** Crude and overlap propensity score-weighted odds ratios for gastric cancer in relation to the treatments for chronic periodontitis within two different time periods from the index date, with subgroup analyses according to age, sex, income, and region of residence.

Characteristics		Odd Ratios for Gastric Cancer (95% Confidence Interval)			
		Crude	*p*-Value	Overlap Weighted Model †	*p*-Value
From the index date to the before the 1-year period					
Total participants (n = 50,870)		1.04 (0.88–1.21)	0.671	1.31 (1.15–1.49)	<0.001 *
	Age < 65 years old (n = 25,705)	1.20 (0.97–1.50)	0.095	1.50 (1.25–1.80)	<0.001 *
	Age ≥ 65 years old (n = 25,165)	0.88 (0.69–1.11)	0.27	1.10 (0.90–1.33)	0.346
	Male (n = 34,170)	1.06 (0.88–1.27)	0.556	1.32 (1.13–1.54)	<0.001 *
	Female (n = 16,700)	0.98 (0.72–1.34)	0.884	1.25 (0.97–1.62)	0.091
	Low-income group (n = 24,200)	1.01 (0.80–1.28)	0.936	1.33 (1.09–1.62)	0.005 *
	High-income group (n = 26,670)	1.06 (0.85–1.31)	0.616	1.29 (1.08–1.55)	0.005 *
	Urban residents (n = 21,550)	1.09 (0.87–1.36)	0.465	1.33 (1.10–1.60)	0.003 *
	Rural residents (n = 29,320)	0.98 (0.78–1.24)	0.891	1.28 (1.06–1.55)	0.009 *
From the index date to the before the 2-year period					
Total participants (n = 50,870)		1.04 (0.94–1.15)	0.456	1.24 (1.14–1.35)	<0.001 *
	Age < 65 years old (n = 25,705)	1.16 (1.00–1.33)	0.049 *	1.38 (1.22–1.55)	<0.001 *
	Age ≥ 65 years old (n = 25,165)	0.93 (0.80–1.08)	0.357	1.09 (0.96–1.23)	0.168
	Male (n = 34,170)	1.05 (0.93–1.19)	0.396	1.25 (1.13–1.38)	<0.001 *
	Female (n = 16,700)	1.00 (0.82–1.23)	0.966	1.20 (1.02–1.42)	0.028 *
	Low-income group (n = 24,200)	1.04 (0.89–1.22)	0.592	1.32 (1.17–1.51)	<0.001 *
	High-income group (n = 26,670)	1.04 (0.90–1.19)	0.602	1.18 (1.05–1.32)	0.006 *
	Urban residents (n = 21,550)	1.07 (0.93–1.24)	0.343	1.26 (1.11–1.42)	<0.001 *
	Rural residents (n = 29,320)	1.01 (0.87–1.17)	0.927	1.22 (1.08–1.37)	0.001 *

* Significance at *p* < 0.05. † Adjusted for age, sex, income, region of residence, and Charlson Comorbidity Index.

## Data Availability

All data were obtained from the database of the National Health Insurance Sharing Service (NHISS) and are available at https://nhiss.nhis.or.kr/ (accessed 15 November 2022). The NHISS allows access to all data (downloaded from the website) for any researcher who agrees to follow the research ethics and pays a processing fee.
